# Rethinking the pathway to sustainable fire retardants

**DOI:** 10.1002/EXP.20220088

**Published:** 2023-07-06

**Authors:** Jiabing Feng, Lei Liu, Yan Zhang, Qingsheng Wang, Hong Liang, Hao Wang, Pingan Song

**Affiliations:** ^1^ China‐Australia Institute for Advanced Materials and Manufacturing Jiaxing University Jiaxing China; ^2^ College of Environment and Safety Engineering Qingdao University of Science and Technology Qingdao China; ^3^ Laboratory of Polymer Materials and Engineering NingboTech University Ningbo China; ^4^ Department of Chemical Engineering Texas A&M University Texas USA; ^5^ Mary Kay O'Connor Process Safety Center, Artie McFerrin Department of Chemical Engineering Texas A&M University Texas USA; ^6^ Centre for Future Materials University of Southern Queensland Springfield Australia; ^7^ School of Agriculture and Environmental Science University of Southern Queensland Springfield Australia

**Keywords:** costs, efficiency, flame retardants, life cycle assessment, sustainability

## Abstract

Flame retardants are currently used in a wide range of industry sectors for saving lives and property by mitigating fire hazards. The growing fire safety requirements for materials boost an escalating demand for consumption of fire retardants. This has significantly driven both the industry and scientific community to pursue sustainable fire retardants, but what makes a sustainable flame retardant? Here an overview of recent advances in sustainable flame retardants is offered, and their renewable raw materials, green synthesis and life cycle assessments are highlighted. A discussion on key challenges that hinder the innovation of fire retardants and design principles for creating truly sustainable yet cost‐effective fire retardants are also presented. This short work is expected to help drive the development of sustainable, cost‐effective fire retardants, and expedite the creation of a more sustainable and safer society.

## Graphical abstract

Rethinking the pathways to sustainable fire retardants, from the perspective of sustainability, costs, and efficiency.

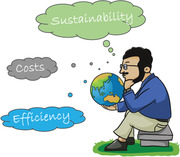

## 1 INTRODUCTION

**FIGURE 1:**
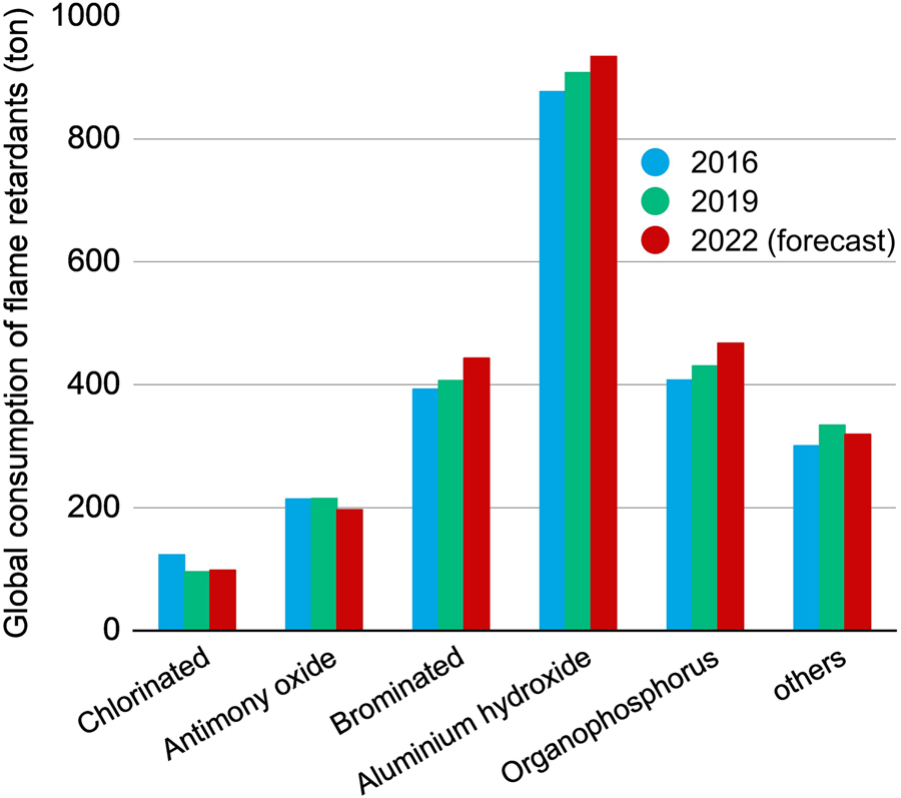
Global production of flame retardants.

Due to the growth of fire safety requirements in end‐use industries, the production of flame retardants (FRs) has become a multibillion‐dollar industry.^[^ ^1^ ^]^ These FRs eventually are applied in sectors including construction, transportation, electronics and wire & cables, as well as textiles. The estimated global consumption of fire retardants (FRs) in 2016 was 2.3 million metric tons and this value is projected to reach ≈2.5 million metric tons (see Figure 1). Among these FRs, aluminum hydroxide accounts for the largest market share (≈38%), and organophosphorus FRs takes the second place, in ≈18%, closely followed by brominated FRs. Currently, halogenated FRs including brominated and chlorinated products, as well as their synergist antimony trioxide are still consumed extensively, but their potential biotoxicity^[^ ^1^, ^2^, ^3^, ^4^, ^5^, ^6^ ^]^ makes them suffer increasing restrictions from regulatory authorities, which catalyzes a greater need for non‐halogenated counterparts such as organophosphorus FRs that are widely accepted as more environmentally friendly alternatives. However, some studies uncovered phosphorus (P)‐based FRs also have persistence and toxicity issues,^[^ ^7^, ^8^ ^]^ so what makes a truly sustainable flame retardant?

In manufacturing, some carcinogenic brominated FRs have been banned by regulatory bodies, but regrettably resultant less harmful substitutions, in many cases, are still bromine‐containing additives because of their higher cost‐effectiveness. This results in the current large consumption of halogenated FRs (including their synergists), and even might drive more demand for them. Worse still, due to the scant information on their potential health and environmental impacts, the brominated alternatives could still be harmful to our enviorment and health. This compromise in sustainability for pursuing profits is not commendable.

In comparison, academics take a step forward towards sustainable FRs. Many bio‐based FRs derived from cellulose, lignin or phytic acid, etc. have been recently developed by scientists.^[^ ^9^, ^10^ ^]^ Renewable feedstock can significantly avoid environmental concerns caused by petrochemical extraction.^[^ ^11^ ^]^ In addition, researchers have reported green synthesis of FRs, for example, aqueous solution and solvent‐less synthetic routes.^[^ ^12^, ^13^, ^14^ ^]^ The elimination of toxic solvents greatly contributes to minimizing environmental hazards and potential worker exposure. Moreover, scientists have started to assess toxicological profiles of some organophosphorus FRs.^[^ ^15^, ^16^, ^17^, ^18^ ^]^ Meanwhile, a few studies on life cycle assessment (LCA) of fire‐retarding polymers were reported in recent years.^[^ ^19^, ^20^, ^21^ ^]^ Until now, for non‐halogenated FRs, there are still inadequate information on their environmental impacts and even toxicity, but these early‐stage investigations have raised a public awareness of the potential chemical risks from the new‐emerging FRs, particularly the P‐based or nitrogen (N)‐containing ones.

**FIGURE 2:**
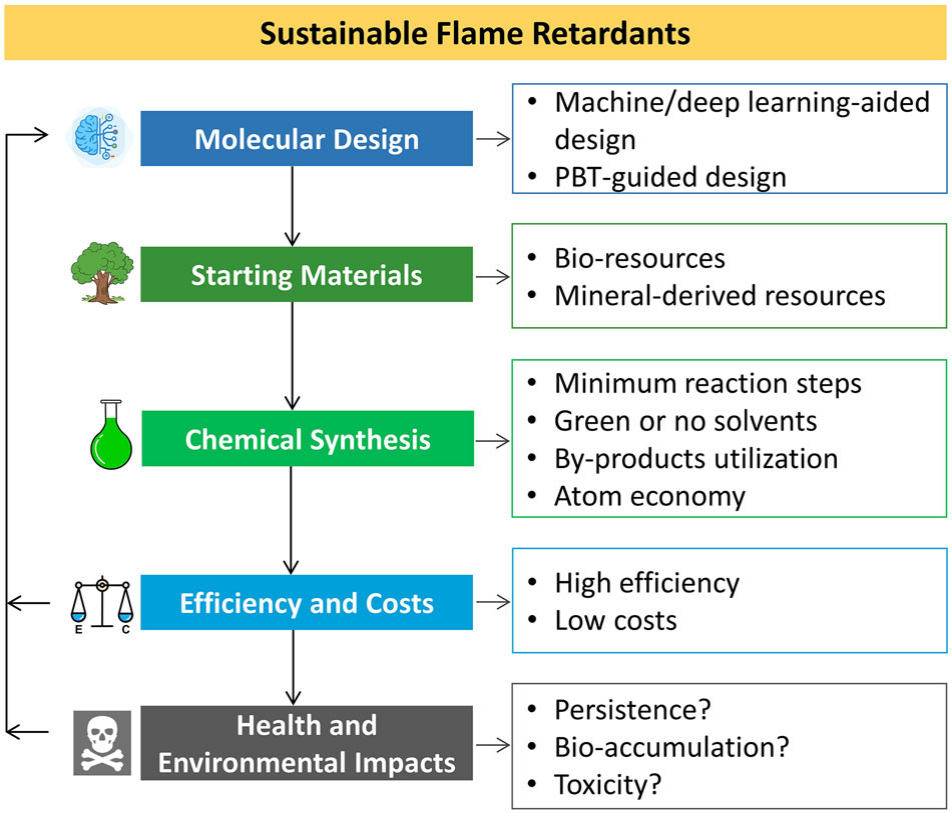
Design principles for sustainable flame retardants.

Despite great advances in the development of sustainable FRs, many challenges remain to be indentified carefully and overcome in a proper manner (see Figure 2):

1. How machine learning guides molecular design of a fire retardant?
2. How to realize a fully nature‐derived fire retardant?
3. What does a green synthetic route look like?
4. Can halogen‐free FRs justify their chemical safety prior to extensive use?
5. How to thoroughly do the LCA of a fire‐retardant product, and can LCA be used for guiding the design of sustainable fire retardants?

Unlike previous perspectives on criticism of halogenated FRs,^[^ ^1^, ^22^, ^23^ ^]^ here we highlight key challenges on the pathway to sustainable FRs, with the entire cradle‐to‐grave lifecycle discussed.

## 2 MOLECULAR DESIGN

The efficiency of a flame retardant is normally determined by its molecular structure and targeted matrix. Current molecular design of flame retardants heavily relies on experience and intuition, thus normally leading to largely trial‐and‐error processes involving large number of chemicals, synthesis, formulas and tests. These empirical investigations might ultimately create desired fire retardants, but surely are not efficient and economical, and even caused a considerable chemical wastes issue. For example, Gaan et al. designed eight different but similar phosphoramides, with 32 fire‐retarding polyurethane foams (PUF) prepared.^[^ ^24^ ^]^ Among these formulas, only one makes PUF achieve a desired UL‐94 HF1 rating but meanwhile consumes many chemicals. Therefore, an advanced methodology toward the molecular design of high‐efficient flame retardants without costly experiments is urgently required.

Machine learning, a promising date‐centric approach, has advanced dramatically over the past decade. More recently, this technology has been successfully applied to the design of gas‐separation membranes,^[^ ^25^, ^26^ ^]^ polyelemental heterostructures^[^ ^27^ ^]^ and plastic depolymerization.^[^ ^28^ ^]^ Although some machine learning algorithms that forecast the flame retardancy of polymers have recently been reported, the prediction models are based on flame retardancy index^[^ ^29^ ^]^ or limiting oxygen index (LOI)^[^ ^30^ ^]^ of materials rather than on the molecular design of fire retardants. Herein, we assume that the molecular design of fire retardants could be aided by a machine (or deep) learning model. Specifically, the combustion properties of a compound (or polymer) are closely associated with its molar group contribution that can be reflected by heat release capacity (HRC) values.^[^ ^31^ ^]^ On the basis of this principle, a self‐taught machine learning mode can be trained by inputting a huge number of HRC values, and then it can accurately predicate specific compounds with high fire‐retardant efficiency. Also, the feasibility of synthesizing the predicated compounds and whether they are matching for targeted matrices need to be fully taken into consideration. Using this machine learning‐aided design method, a highly effective realizable flame retardant tailored toward a specific polymer matrix can be obtained while avoiding high‐cost trials.

As a result, the machine learning algorithm has great potential for the molecular design of high‐efficient fire retardants, but meanwhile more databases regarding HRC values and steric effect of chemical groups should be established and optimized to make this promising method achievable.

## 3 STARTING MATERIALS

Existing raw materials for synthesizing organic FRs in industry are nearly petrochemicals that are generally refined from finite fossil resources. The petrochemical extraction has caused some environmental issues such as climate change and pollution.^[^ ^11^ ^]^ For these reasons, renewable resources are more encouraged to be raw materials of FRs.

**FIGURE 3:**
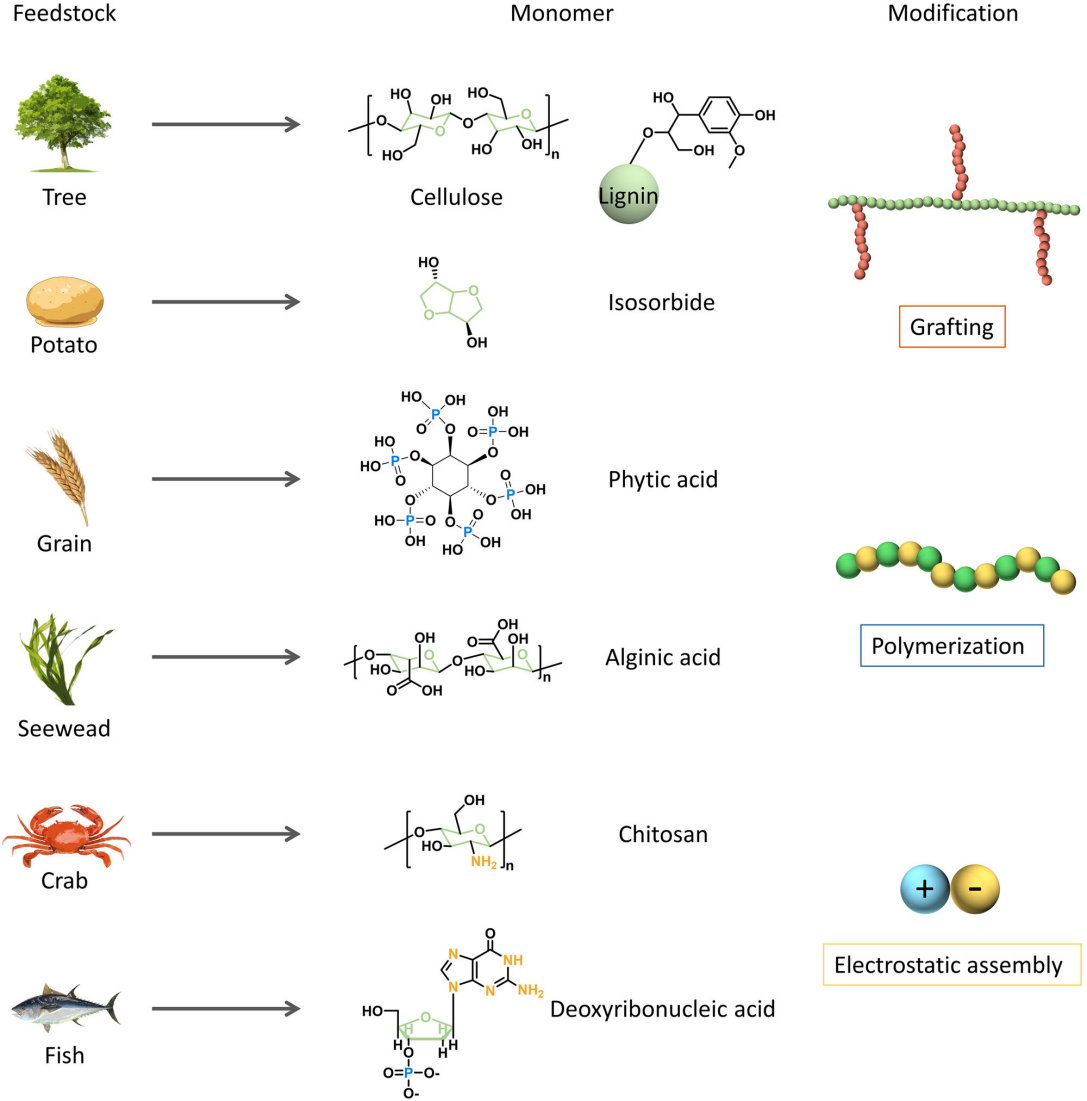
Natural feedstock as raw materials for sustainable flame retardants.

Plant‐derived feedstocks including cellulose, lignin, phytic acid and algin, etc. (see Figure 3) have increasingly attract attentions as starting materials of FRs in the past few years due to their abundant reserves and eco‐friendliness. Cellulose, the most abundant biopolymer in nature, has exceptional charring ability, making it a green carbon source. An all‐in‐one cellulose‐based intumescent flame retardant (IFR) was recently reported as an effective flame retardant for papers.^[^ ^32^ ^]^ Lignin, the second abundant biomass after cellulose, also has emerged as a potential source for FRs. It has been reported that lignin‐derived FRs can improve the fire resistance of polypropylene.^[^ ^33^, ^34^ ^]^ Recently, more lignin‐based FRs were developed for bioplastics.^[^ ^35^, ^36^, ^37^ ^]^ In addition to achieve a high value‐added utilization of lignin waste, these work furthers the development of both bio‐based FRs and fire‐retarding bioplastics.

Phytic acid mainly exists in plant seeds and it also has been reported as raw materials for bio‐derived FRs.^[^ ^38^, ^39^, ^40^, ^41^ ^]^ Among them, a green electrochemical approach for a hybrid of ferric phytate and graphene is very interesting, in which phytic acid plays a role as an electrolyte and a modifier, simultaneously. This ferric phytate functionalized graphene results in a significant suppression on the fire hazards of polylactide (PLA), as reflected by a 40% reduction in peak hear release rate (pHRR).^[^ ^40^ ^]^ Isosorbide can also be derived from plants (such as potatoes), and modified isosorbide compounds are effective in improving the fire retardancy of PLA or polybutylene succinate (PBS).^[^ ^42^, ^43^ ^]^ Another biomass resource, alginic acid, is mainly derived from brown algae and its salts, such as calcium alginate has been demonstrated to present excellent fire retardancy,^[^ ^44^, ^45^ ^]^ thus making alginic acid a green candidate for bio‐based FRs.

In addition to plants, animal‐derived substances can be another natural source for creating bio‐based FRs. Chitosan is a sugar that comes from the outer skeleton of shellfish including crab, lobster and shrimp. It was very often assembled with other negative charged compounds such as phytic acid and alginate via layer‐by‐layer technique.^[^ ^46^, ^47^, ^48^, ^49^ ^]^ This combination between/among biomolecules dramatically promotes the sustainability of fire retardants. Other bioresources, such as deoxyribonucleic acid (DNA), can also be extracted from animals. DNA has recently presented a great potential as a raw materials of FRs because of its all‐in‐one IFR feature.^[^ ^9^ ^]^

Mineral resources extensively exist in nature. Although they are unrenewable, the utilization of them can help reduce carbon footprint. Currently, many FRs are derived from minerals. Aluminum hydroxide, the largest single flame retardant at 38% market share, is produced from bauxite. In scientific research, montmorillonite (MMT) and halloysite, came from smectite and kaolinite respectively, were consistently reported as raw materials of FRs over the two decades.^[^ ^50^, ^51^, ^52^, ^53^ ^]^ Their unique layered or tubular nanostructure endows polymer matrices with enhanced thermal and/or mechanical properties, in addition to excellent suppression on heat release. Recently, sepiolite and vermiculite were also used to create fire‐retardant coatings.^[^ ^54^, ^55^, ^56^, ^57^ ^]^ They enable the substrates to show reduced flammability and lower smoke production. Therefore, minerals represent a promising source of green FRs, and more economical mineral‐based FRs are strongly anticipated to replace low‐efficient aluminum hydroxide.

Many progresses in the development of natural derived fire retardants have been made in academia, but some challenges still remain. Primally, nearly all the natural resources, such as cellulose or MMT, need to be chemically modified via phosphorylation and/or physically introducing traditional nitrogen components (e.g., melamine) to achieve desired flame retardancy or better compatibility with polymer matrices prior to use. The incorporation of traditional petrochemicals compromises the natural feature of these fire retardants to some extent. In addition, the toxicity of these biomolecules or minerals is seldom considered because they are unintentionally regarded as unharmful chemicals. In fact, natural‐derived molecules might be not harmful to the environment, but are likely to be toxic (e.g., for phytic acid, its median lethal dose (LD_50_) to male rat (oral) is 405 mg kg^−1[^ ^58^ ^]^). Their final challenge is that the relatively high price of bioresources limits their practical use  due to expensive biorefining processes.

Recently, industry waste was reported as raw materials for FRs. For example, steel slag powder can improve the flame retardancy of rigid PUF when combined with Melamine pyrophosphate (MPP).^[^ ^59^ ^]^ The results show that these thermally stable inorganic substances can enhance the thermal resistance of the char promoted by MPP, thus contributing to higher LOI values and lower pHRR values as compared with the performance when the use of MPP alone. But still the same problem, the steel slag itself often shows very limited fire retardancy efficeincy . As a resut, it needs additional modifications or combination with other organic FRs to boost its flame retardancy effciency

To obtain more sustainable but economic FRs, a low‐cost biorefining technology targeting for biomolecules is necessarily required. More nontoxic biochemicals (P or N compounds are more preferred) and minerals need to be available to support the development of fully nature‐derived flame retardants. Also, for encouraging the utilization of resources, some industry wastes, such as steel slag, are recommended to be combined with biomass FRs for achieving higher cost‐effectiveness.

## 4 CHEMICAL SYNTHESIS

Synthetic pathways are vital for the yield, costs, and even sustainability of FRs. Among them, green synthetic routes require more attention because current production of FRs usually involves complicated reactions, toxic solvents and/or chemicals waste. This kind of production has not only led to higher process costs but also caused environmental issues, including carbon emissions and industrial waste. For these reasons, more facile but eco‐benign synthetic pathways are urgently needed for promoting the sustainable manufacturing of FRs.

Simplification of synthetic routes of flame retardants will contribute to reduction in process costs and carbon footprint. When selecting a reductionist synthetic approach, less chemicals, including reagents, catalysts and solvents, involve, which minimizes the risks of worker exposure and reduces industrial waste.^[^ ^60^ ^]^ Unfortunately, many existing synthesis methods of FRs often involve multi‐step reactions to achieve desired chemicals. Typically, some ammonium polyphosphate (APP) derivatives^[^ ^61^, ^62^ ^]^ were synthesized by three or more reaction steps. In addition to increased manufacturing costs, a complex synthetic pathway will bring more uncertainties in chemical risks (e.g., waste disposal, chemical leakage and even explosion), as well as consequential environmental issues. In brief, the synthetic routes of FRs should be simplified to forward their sustainability.

To alleviate possible chemical risks from toxic organic solvents (e.g., dichloromethane and toluene), green solvents synthesis attracts growing attention when being used for preparing FRs. Using no‐toxic solvents to synthesize fire retardants largely reduces harmful impacts to human and the environment. Recently, aqueous solution is applied in preparation of core‐shell bio‐based FRs. The synthesis was successfully conducted via layer‐by‐layer (LbL) assembly in the water medium, demonstrating a green strategy for creating  FRs.^[^ ^46^, ^48^ ^]^ However, the LbL assembly  often encompasses multi‐step reactions, thus compromising the production efficiency. An one‐step ion exchange reaction in aqueous solution was developed by Zhang et al.^[^ ^63^ ^]^ to synthesize an IFR. This work demonstrates a green yet efficient pathway for creating FRs.

Recently, solvent‐free synthesis approaches have also been developed. Early in 2004, Watterson et al. reported a solventless enzyme‐mediated polymerization that developed an inherently fire‐retardant siloxane‐based polymer.^[^ ^64^ ^]^ More recently, Feng et al. reported a one‐step solvent‐free approach for synthesizing polyphosphoramide fire retardants that endow PLA with excellent flame retardancy.^[^ ^12^, ^13^, ^14^ ^]^ For this green yet facile synthesis strategy, no post‐treatment is involved, including products purification and solvent waste disposal. For these eco‐friendly advantages, a growing number of attempts focusing on green solvents or solvents‐free methods for FRs synthesis should be encouraged, expediting the real‐world application of these green approaches in industry.

Another issue in synthesis routes is recycling of by‐products. Most synthesis of organic P‐, N‐ or P/N‐containing FRs produces by‐products owing to the substitution reaction. Typically, both the synthesis of (poly)phosphonates^[^ ^65^, ^66^ ^]^ and (poly)phosphoramides^[^ ^67^, ^68^, ^69^ ^]^ involves triethylamine hydrochloride. This chemical waste is worthy of recycling rather than disposal as a waste given that it is a basic raw material for quaternary ammonium salts, medicines and dyes. Another example is methanol formed in the green synthesis of polyphosphoramides based on amine ester exchange reaction.^[^ ^12^, ^13^, ^14^ ^]^ The only by‐product methanol can be collected via vacuum pumping system and reused. Thus, it is advisable to make more efforts to convert the chemical waste to resources.

In comparison to reuse of by‐products, atom economy should be more advocated in FRs synthesis. Fortunately, addition reactions make it possible with less or even no by‐products. 9,10‐dihydro‐9‐oxa‐10‐phosphaphenanthrene10‐oxide (DOPO) offers many examples, in which it reacts with alkenyl‐containing triazine compounds via addition reactions.^[^ ^70^, ^71^, ^72^ ^]^ This strategy significantly forwards the practice of minimizing waste to the molecular level, making atoms present in the starting materials and end up in the products rather than in the waste.

The green synthetic routes in pursuit of a simple reaction, green (or no) solvent and waste recycling, as well as atom efficiency, will surely propel the development of green flame retardants. They do offer bright solutions in the area of toxicity reduction and waste prevention. Although some progresses in green synthetic strategies have been made, more attempts on them should be encouraged both in academia and industry, to drive these green synthetic technologies to real‐world applications.

## 5 EFFICIENCY AND COSTS

Efficiency is a key issue for FRs. A high loading of flame retardants additives usually leads to adverse impact on physical properties (e.g., mechanical performance and thermal stability) of materials bulk. Thus, the scientific community never stop striving for higher fire‐retardant efficiency. Wang's group reported a phosphoramide compound (P‐AA) that is extremely efficient for PLA. only addition of 0.3 wt% P‐AA make PLA pass a UL‐94 V‐0 rating (3.2 mm).^[^ ^73^ ^]^ Very recently, an in situ crosslinking P/N flame retardant (PBD) shows a very high efficiency in epoxy resin. With only addition of 0.5 wt% PBD, epoxy can achieve a UL‐94 V‐0 rating and a high LOI of 32.1%.^[^ ^74^ ^]^ These advances do offer a promising way to realize desired fire safety of materials yet preserve their bulk properties, but more advanced artificial intelligence (AI) technologies, such as machine learning, are certainly worthy of developing to help researchers design high‐efficient fire retardants.

The price of a fire retardant is a key factor that determines whether this product will be acceptable in the market. However, the costs of preparing a flame retardant are often unintentionally ignored by researchers because they value its fire‐retardant properties more than production costs. This makes the fire retardant production difficult to be scaled up. In fact, the costs control can be prioritised in the phase of raw materials selection. Some commercially available flame retardants, such as APP, DOPO and melamine are cost‐effective choices to develop new FRs. Moreover, the easily accessible biomass including phytic acid and cellulose should be advocated not only for their relatively low cost but also for the sustainability. Synthesis costs is another factor in derterminingde the final market price of a flame retardant. As mentioned above, reducing synthetic steps and avoiding waste from solvents or by‐products are promising ways to lower production costs.

Briefly, more factors affecting the costs of FRs are required to be taken into consideration when designing them. The revolution in sources and synthetic routes is anticiated to further lower the price of flame retardants . Also, more comprehensive evaluation methods of fire retardancy efficiency should be established.

## 6 HEALTH AND ENVIRONMENTAL IMPACTS

In addition to renewable raw materials and eco‐benign synthesis, a truly green flame retardant must not be harmful to human health and the environment. The criticism against chemical risks from halogenated flame retardants has never been stopped,^[^ ^1^, ^5^, ^22^ ^]^ and this makes them partly banned by regulations, but many substitutions still contain bromine. De Boer and Stapleton considerer this as ‘repeated regrettable substitutions’ because scant information was available on their long‐term toxicologic effect.^[^ ^1^ ^]^

Over the past decade, researchers started to draw attention to toxicological profile of non‐halogenated flame retardants, such as aluminum diethylphosphinate (ADP) and DOPO derivatives.^[^ ^7^, ^8^, ^17^ ^]^ But a publication funded by ENFIRO, a project targeting at assessment of environment‐compatible flame retardants, pointed out that most halogen‐free alternatives still are short of information on their environmental behaviors and toxicological impacts.^[^ ^75^ ^]^ Also, the current toxicological data of flame retardants obtained from short‐term tests is still not enough to unfold their real profiles. Unfortunately, manufacturers still choose to regard halogen‐free flame retardants as low‐toxic or even green chemicals. Worse still, more novel P‐ or N‐containing fire retardants were synthesized and reported by academics without proper risk assessments.

Another issue is the current not well‐developed evaluation method on the basis of persistence, bio‐accumulation and toxicity (PBT). The first problem is the minimal consideration given to transformation compounds when assessing the persistence. In fact, the transformation compounds maybe more harmful than the parent flame retardant. For this reason, persistence and toxicity of the transformation chemicals should also be investigated when discussing the PBT issue of a fire retardant, rather than just focus on the parent compounds. Encouragingly, Liu and co‐workers recently noticed this issue and tried to fill this knowledge gap. They developed a new framework applied in organophosphate flame retardants, and they found the transformation products are globally distributed across 18 megacities. The authors claimed that this is a previously unrecognized exposure risk for the world's urban populations.^[^ ^76^ ^]^

In a word, more collaboration among chemists, toxicologist and ecologist are encouraged in FRs (particularly non‐halogenate ones) investigations, which can help provide more long‐term information on their environmental and toxicological profiles. Meanwhile, a well‐developed framework for risks assessment should be established before their large‐scale production and use. More importantly, scientists and engineers are strongly encouraged to use the PBT assessment results to guide the molecular design of low‐toxic FRs. In this way, harmful FRs would not be accessible to the end‐use industry, and truly green FRs can replace the current problematic ones.

## 7 LIFE CYCLE ASSESSMENT OF FIRE‐RETARDANT PRODUCTS

**FIGURE 4:**
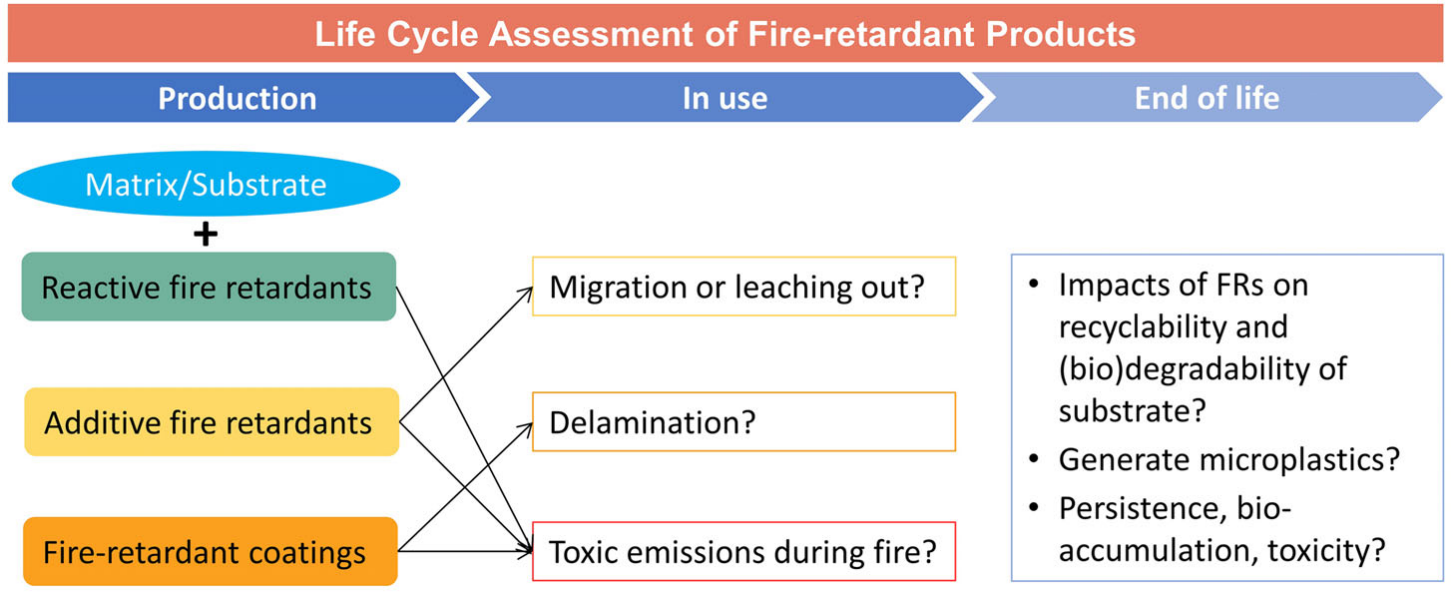
Life cycle assessment of fire‐retardant products focusing on the impacts of fire retardants.

LCA of FRs should not be limited to FRs themselves because they finally end up existing in various products, such as furniture, electronics. Some new environmental issues may arise due to interactions between additives and matrices by somehow. Thus, a critical risk assessment of fire‐retardant products but more focusing on the health and environmental impacts of FRs are required (see Figure 4). Although some life cycle assessments of fire‐retardant products including electronics were carried out,^[^ ^19^, ^21^ ^]^ more challenges have not been overcome or even never been taken in account.

The first question is how easy it is for fire retardants to enter the environment and/or human bodies from products. Additive‐type FRs, especially some molecular weight compounds, are very like to leach out from matrices. Fire‐retardant coatings also have chances to access to the ecosphere because of delamination. Many measures were taken to try to solve the exposure risk. Some polymeric flame‐retardant additives were used to prevent the potential migration issue. Typically, a brominated polybutadiene‐polystyrene flame retardant is commercially applied in expanded polystyrene products to reduce the human exposure to flame retardants, but it still remains a global concern because of the presence of bromine.^[^ ^23^ ^]^ As for the novel P‐based polymeric fire retardants developed in academia, such as polyphosphates and polyphosphoramides, regrettably their migration behavior has not yet been critically assessed. For the delamination of coatings, some progresses were made to achieve strong adhesion with substrates,^[^ ^77^, ^78^ ^]^ but these results were obtained through short‐term testing and whether the coatings delaminate or not during the phase of ‘in use’ still remains unknown.

All fire‐retarding products, including those containing reactive additives, have the risk in toxic emissions during an accidental fire, not only because of the materials bulk, but also because of the flame retardants themselves. It has been widely accepted that most non‐halogenated flame retardants perform better in smoke suppression and generates less hazardous gases relative to halogenated FRs when they are burned. But the P‐, N‐ or P/N‐based FRs still produce possible toxic gases, such as phosphite, and nitric oxides. Additionally, is there a possibility for halogen‐free flame retardants to react with their matrices during a fire and then forms more harmful smoke? Therefore, we need to dig into the details of the smoke toxicity of halogen‐free flame retardants, as well as their fire‐retardant products.

The impacts of flame retardants still exist even after the disposal of the products. The first challenge is the treatment of fire‐retardant products. The addition of fire retardants (especially in high loading) makes it difficult to achieve bulk materials recycling, presenting a clear disadvantage in the circular economy. For the newly emerging biodegradable polymers such as PLA, there is very few information on how the fire retardants affect their biodegradability. A recent study has drawn attention to this issue. A bio‐based fire retardant (PA@CHTM) can accelerate the degradation of PLA in soil,^[^ ^49^ ^]^ which is an exciting starting that is expected to encourage more investigation on the biodegradable behavior of biopolymers.

If the products are treated by landfilling, the persistence of fire retardants needs to be reconsidered because their chemical structures can change significantly when they exist in/with a particular polymer matrix or substrate. The first unknown is whether a fire retardant will be more persistent when it exists with microplastics formed from the matrix even if it is easy to degrade by itself.^[^ ^79^ ^]^ Then, will a polymeric fire retardant break down into microplastics and finally result in the formation of microplastics mixtures. These concerns have not been taken seriously, but it definitely would be a disaster if more harmful microplastics enter into the environment.^[^ ^80^, ^81^, ^82^, ^83^ ^]^

To obtain sustainable fire retardants, on one hand, critical LCA methods should be developed. More investigations on the leaching and deamination problems and the potential risks brought by them should be undertaken. On the other hand, scientists can consider the LCA results as database to guide the design more sustainable FRs.

## 8 OUTLOOK

In this paper, we provide a review of main achievements in developing sustainable FRs, and present a discussion on the key challenges that need to be addressed for realization of truly sustainable FRs, with the entire cradle‐to‐grave life of FRs critically considered. Primally, if as suggested, a machine learning‐aid molecular design method will be helpful. By learning from the current database based on HRC values of groups, the AI technology can accurately predict a molecular structure that will exhibit high fire‐retarding efficiency. Also, the subsequent feedbacks from efficiency evaluation and PBT assessments, as well as LCA of fire‐retarding products can be relearnt and reprocessed by the machine learning model, and then it can help accelerating the creation of more sustainable and higher efficient FRs.

Nature‐derived FRs have recently attracted attentions, but most renewable compounds or natural minerals need to be chemically modified by petrochemicals prior to use. Thus, more efforts are required to develop biorefining technologies to obtain more cost‐effective biomolecules that can propel the realization of fully natural‐derived FRs. Meanwhile, green synthesis is often undervalued. Chemists should be involved more in the pathway design actively and offer more reductionist approaches. More investigations into green or no solvents synthesis are necessary to make sustainable FRs, with the collection and reuse of by‐products taken into account. Atom economy is also highly recommended to maximize the atom efficiency and achieve waste prevention at the molecular level.

Concerns over the potential PBT issue of current FRs still remain and terribly there is scant information on their environmental and toxicological profiles, especially for halogen‐free FRs. Another issue is the current problematic PBT assessment method. The transformation compounds derived from FRs need to be carefully considered when one evaluating PBT. For LCA of fire‐retarding products, we appeal for more consideration referring FRs should be given, not only to possible toxic emissions from FRs during a fire, but also the fate of FRs, as well as their persistence and toxicity after disposal of the fire‐retardant products. Close collaborations among chemists, toxicologist and ecologists, as well as AI experts, are imperatively needed to make a thorough and long‐term chemical risk assessment of FRs. The subsequent assessment results can be reprocessed by the AI technology, finally guiding to sustainable FRs.

Some aspects regarding sustainability of FRs lie beyond the scope of this study. We do believe engineers in flame retardants industry will find a way to achieve the aim of sustainable yet cost‐effective FRs, but before that happens, there is still a lot of work ahead for scientists to do to clear the roadblocks on the pathway to sustainable FRs. We hope that this study will motivate further work focusing on the development of sustainable FRs that will be hopefully realized in real‐world applications.

## CONFLICT OF INTEREST STATEMENT

The authors declare no conflicts of interest.
